# A pilot study on preferences from surgeons to deal with an innovative customized and connected knee prosthesis – A discret choice experiment

**DOI:** 10.1016/j.heliyon.2024.e30041

**Published:** 2024-05-08

**Authors:** Mathieu Le Stum, Arnaud Clave, Koffi Adzinyo Agbemanyole, Eric Stindel, Myriam Le Goff-Pronost

**Affiliations:** a Université de Brest, UBO, LATIM, UMR 1101, 22 rue Camille Desmoulins, 29200, Brest, France; b Institut National de la Santé et de la Recherche Médicale, Inserm, LaTIM, UMR 1101, 22 rue Camille Desmoulins, 29200, Brest, France; c Service d'orthopédie, Clinique Saint George, 2 Avenue de Rimiez, 06100, Nice, France; d Institut Mines-Telecom, IMT Atlantique, LATIM, UMR 1101, M@rsouin, 655 Av. du Technopôle, 29280, Plouzané, France; e Centre Hospitalo-Universitaire de Brest, CHU Brest, LATIM, UMR 1101, 2 Avenue Foch, 29200, Brest, France

## Abstract

**Background:**

To address the increasing global demand for Total Knee Arthroplasty and reduce the need for revisions, several technologies combining 3D planning and artificial intelligence have emerged. These innovations aim to enhance customization, improve component positioning accuracy and precision. The integration of these advancements paves the way for the development of personalized and connected knee implant.

**Questions/purposes:**

These groundbreaking advancements may necessitate changes in surgical practices. Hence, it is important to comprehend surgeons' intentions in integrating these technologies into their routine procedures. Our study aims to assess how surgeons' preferences will affect the acceptability of using this new implant and associated technologies within the entire care chain.

**Methods:**

We employed a Discrete Choice Experiment, a predictive technique mirroring real-world healthcare decisions, to assess surgeons' trade-off evaluations and preferences.

**Results:**

A total of 90 experienced surgeons, performing a significant number of procedures annually (mostly over 51) answered. Analysis indicates an affinity for technology but limited interest in integrating digital advancements like preoperative software and robotics. However, they are receptive to practice improvements and considering the adoption of future sensors.

**Conclusions:**

In conclusion, surgeons prefer customized prostheses via augmented reality, accepting extra cost. Embedded sensor technology is deemed premature by them.

## 1 Introduction

Total Knee arthroplasty (TKA) is one of the most predominant orthopedic procedures worldwide with respectively 720,000 in 2019 and more than 700,000 surgeries in Europe [1] and the USA [2]. This number is continuously increasing for several decades and is expected to explode in the next years (+147 % by 2030 in the USA [2]).

This overall increase in TKA is chiefly driven by the natural rise in the incidence of osteoarthritis, related in particular to the combined effects of population aging and obesity [3]. Nonetheless, another factor is the broadening of TKA indications towards younger patients (<65 years) allowed by improvements in both surgical techniques and knee implants [4,5]. As a result, implanted for more than 20 years in active subjects, these prostheses are more frequently revised, leading to expensive and less effective procedures. A +182 % growth in revision Knee Arthroplasty is, for example, expected in the USA by 2030 [6].

To address this major public health problem and improve primary TKA surgery, thereby preventing revisions caused by premature wear, loosening, and infection, several factors must be considered: implant design, surgical technique, and patient follow-up [[7], [8], [9]] [[7], [8], [9]] [[7], [8], [9]].

Since the last decades, implant design has been improved by the use of advanced technologies mixing 3D planning and artificial intelligence. This led to the development of customized implants, suitable to patient morphology thanks to dedicated CT images [10,11]. To improve accuracy and precision in components positioning, several assisted surgical technologies have been developed, such as navigation, robot, or Personalized Surgery Instrument (PSI) [10,[12], [13], [14], [15]]. Among these new technologies, Augmented Reality (AR) is expanding and appeared to be a promising technology [16,17]. At the same time, to provide not only therapeutic benefits but also follow-up capabilities, different sensors types have been developed [18]. These are smart electronic devices that measure physical properties such as pressure, cinematic, or temperature, and send information to an electronic processor [8]. The ability of sensors to measure several types of data allows them to be used in the surgery itself or during the postoperative patient follow-up. At present most stay in a research state but some technologies start to be implanted [19].

The integration of these improvements will enable the development of personalized and connected implants, as proposed in FollowKnee project [20]. However, these advancements also represent a breakthrough, and the implementation of this new care chain may necessitate adaptations and changes in surgical practices. Therefore, it is crucial to understand surgeons' intentions regarding the adoption of these technologies as part of their routine practice. By considering their preferences, we can better assess the impact of these technologies on patient care.

Thus to evaluate the trade-off made by surgeons, we conducted a Discrete Choice Experiment, which is a technique that is predictive of choices, and mimicked real-world decisions in healthcare decision-making, to elicit preference [21].

Our study aims to assess how surgeons' preferences will affect the acceptability of using this new implant and associated technologies within the entire care chain. To the best of our knowledge, no study on surgeons' choices has been reported thus far.

## 2 Materials and METHODS

### 2.1 Study design

Discrete Choice Experiment (DCE) represents a quantitative stated preference technique for eliciting individual preferences [22]. This method relies on the execution of a survey employing a structured questionnaire tailored to ascertain the relative significance of attributes within the domain of services and healthcare programs. Its principal objective is to elucidate the trade-offs individuals are prepared to undertake between attribute levels to optimize their utility within the spectrum of available alternatives. Since utility is not directly observable by the analyst, it is inferred from the choices made by respondents among the various alternatives presented to them [23].

This method explores, for a specific good or service, the importance of trade-offs that individuals make among attributes and their alternatives using a sequence of choice sets depicting hypothetical scenarios. In practice, respondents are presented with various options or scenarios representing different levels for the same good, aiming to collect their preferences. It is well known that individuals tend to choose options that maximize their utility. Therefore, knowing the utility derived from the consumption of a good or service helps increase individuals' adherence to them [23]. The DCE method has the advantage of being able to simulate existing goods or services as well as eliciting preferences and values for goods or services that do not yet exist and are therefore hypothetical. DCE is thus one of the most widely employed methods for understanding the factors that influence choice. The DCE is suitable for examining our research question. While other preference elicitation methods offer similar potential, they come with drawbacks, such as increased complexity for both analysts and participants, and a higher susceptibility to biases (e.g., Time Trade-off and Standard Gamble), or a reduced fidelity to real-world scenarios (such as ranking and evaluation methods) [24]. Consequently, we have opted for the DCE in this study. This approach is regarded as the most fitting one meeting the requirements of welfare theory [25], as it involves individuals in decision-making processes akin to those encountered in their daily lives.

In our experiment, a DCE was administrated to French surgeons (1) via an online survey, distributed through the French Orthopaedic National Society (Sofcot) and (2) through a regional congress (SOO- Western Orthopaedic Society), from January to June 2022.

The survey takes 10 min to complete and was divided into five sections: (i) sociodemographic (ii) information on current surgical practice, (iii) preference, on the use of an innovative prosthesis, (iv) potential development of practice (i.e. What conditions would you require for the use of a personalized and connected prosthesis?), and (v) affinity for technology interaction (ATI). The survey was designed collaboratively with specialists in both the humanities and social sciences, as well as surgeons. The latter group aided in identifying any inconsistencies in the wording of the questions pertinent to the surgical field.

In the Preferences section, surgeons select their favorite scenario from a series of two hypothetical but realistic scenarios for a customized, connected prosthesis. They were asked in each to select the one they would prefer. The scenarios were described by a set of attributes which were further specified by levels.

To define attributes, good research practices for stated-preference studies [26] were followed. Attributes (Table 1) were identified by a literature review and findings from interviews with representative experts (four orthopedic surgeons, a cognitive scientist specializing in technology adoption, and a rehabilitation physician). It was pointed out the importance of (1) the care pathway before and after the surgery, (2) anatomical planning, (3) precision of the prosthesis placement during the surgery, (4) post-surgery data and their usability, and (5) cost. The full questionnaire was pretested on eight representative orthopedic surgeons to assess their understanding of its content and gather feedback on the questions. The results led us to consider three technical attributes and one related to cost. The technical attributes are “Customized implant” (link to previous point 2), “integrated sensors” (points 1 and 5), and usage of “Augmented reality” (point 3). All the potential use of an innovative customized and connected prosthesis is thus covered. The next step consisted of assigning levels to each attribute. The literature recommends that these should be realistic, well-defined, plausible, and should potentially involve trade-offs. All technical attributes were dichotomous to make able the expression of use, or not, in the current practice. As new technologies often led to an increase in cost, a relating attribute was defined by a low (“+10 %”) or moderate (“+25 %”) rise.

**Table 1:** Attributes and level included in the Discrete Choice Experiment.

Attribute	Definition	Level
Customized Prosthesis	Prosthesis fitting to patient's morphology	Yes
		No
Prosthesis including sensors	Prosthesis integrating pressure, movement, temperature and pH sensors. Data will allow a better post-operation follow-up in order to avoid retrievals (due to wear, loosening and infections)	Yes
		No
Augmented Reality	Use of augmented reality in the operating room to improve accuracy of the prosthesis placement	Yes
		No
Extra costs	If a clinical improvement is proven, an innovative prosthesis would have a possible additional cost for the health system and also for you in terms of training, investment, software …	+10 %
	How much extra cost (€) would you be willing to accept for yourself due to the use of an innovative prosthesis?	+25 %

Data were gathered anonymously. Because our study did not involve the collection of health or personal information, ethics committee approval was not required.

### 2.2 Experimental design

The four attributes with two levels have a possible 2^4^ = 16 scenarios (four attributes with two levels) and (16*15)/2 = 240 possible pairwise choices. Since there are too many possible scenarios, it is impossible to present this full factorial design to the surgeons. Therefore, to simplify the scenario settings, a fractional plan (i.e. selection of the best interaction combinations) was developed that maximizes D-efficiency. D-efficiency is a standard measure of goodness of fit. It indicates how well the main effects can be estimated and retained. A D-efficient design (minimizing D(B) error) was generated by using the R package Idefix to reduce the number of choice scenarios into a manageable number of 16 choice sets for presentation [27,28].

**Fig. 1:**
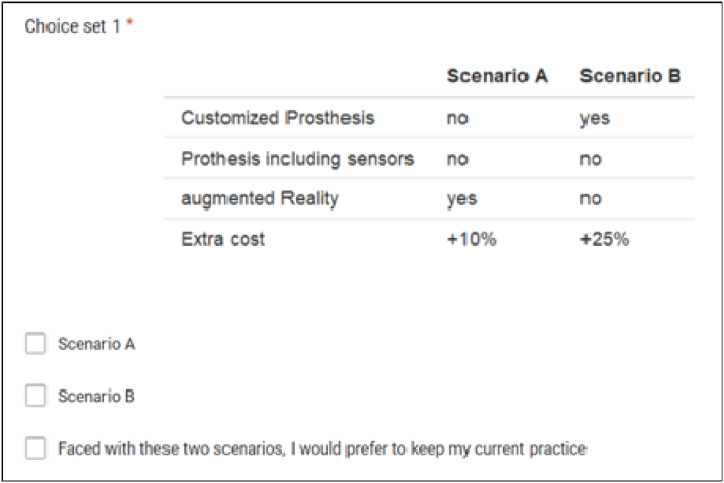
Example of a DCE choice set. DCE, discrete choice experiment.

For most realistic results, it is recommended to stay as close to the real world as possible. When modeling the potential adoption of a new intervention or service, which is a key objective of this paper, there is much consensus that a DCE should include an opt-out option, that figures out a reference point, or their current situation [[29], [30], [31], [32]]. In this study, surgeons are presented with two generic alternatives and an opt-out option (Fig. 1). Opt-out was included to not force the surgeons to choose between the proposed alternatives when those were not considered suitable compared to their current practice. The opt-out option did not describe an alternative model of practice but encompassed all other actual well-proven technologies.

### 2.3 Study size

As there is no consensus to calculate the minimum require sample size in DCE, we used a common rule of thumb [33]. The minimum sample size (N) required for the experiment is thus obtained by formula (1):

(1) N>(500*c)/(t*a)

In which c = equal to the largest number of levels for any of the attributes, t = number of choice set and a = number of alternatives). This results to a minimum sample size of 31 respondents.

### 2.4 Statistical model and data analysis

The answers were analyzed in accordance with McFadden's Random Utility Theory (RUT) (1974), which incorporates economic rationality and utility maximization. Utility is thus not directly derived from the good taken as a whole, but rather derived from each attribute of the good [34]. Accordingly, the RUT posits that the utility gained from selecting a good exhibits a probabilistic nature (McFadden, 1974), with only the deterministic component of this utility being observable to researchers [35]. In other words, individual preferences are influenced by a set of characteristics, among which some are indirectly observable and specific to individuals, while others are random and therefore unobservable. So, according to RUT, each respondent (n) choses their preferred (utility-maximizing) scenario across the three alternatives in each of the 16 choice sets. The utility function (2) as described in Refs. [36,37], can be specified as:

(2) $$U_{\text{nj}}=V_{\text{nj}}+\varepsilon_{\text{nj}}$$

where Vnj is the observable component and εnj is a non-explicable error term (independent and identically distribute). With k attributes, we have the following functional form (3):

(3) $$V_{\text{nj}}=\beta_{1}X_{\text{nj}1}+\beta_{2}X_{\text{nj}2}+\ldots +\beta_{k}X_{\text{njk}}=X{’}_{\text{nj}}\beta$$

And so (2) can be rewritten as:

(4) $$U_{\text{nj}}=X’\text{nj}\beta_{n}+\varepsilon_{\text{nj}}$$

where Unj is the utility that respondent n assigns to alternative j; Xnj, a vector of the observed characteristics of the alternatives (levels); and βn, the vector of coefficients assigned to each level (reflecting the desirability of the attributes) (4).

It is assumed that the form of the function is common across individuals within the sample, but that parameters vary across individuals. To account for heterogeneity in preferences and responses within the surgeons, a mixed logit model (MXL) was used. The MXL identifies attributes for which there is significant preference variation without explaining this variation in depth.

In consequence, the β_n_'s random coefficients can be decomposed into two part, as follows (5):

(5) $$\beta_{n}=\bar{\beta }+\eta_{n}$$

Where $\bar{\beta }$ is the population mean, and η_n_ is a stochastic deviation representing preference heterogeneity. Thus, equation (4) can be rewriten as (6):

(6) $$U_{\text{nj}}=X{’}_{\text{nj}}\;\bar{\beta }+X{’}_{\text{nj}}\;\eta_{n}+\varepsilon_{\text{nj}}$$

The stochastic portion of utility (i.e., X'_nj_ η_n_ + ε_nj_) is correlated across choice situations due to the common influence of η_n_.

In our model, the choice among alternatives depends on four attributes. The utility function to be specified is thus (7):

(7) $$U_{\text{nj}}=\beta_{0}+\beta_{1n}.{\text{Personalization}}_{j}+\beta_{2n}.{\text{Sensors}}_{j}+\beta_{3n}.\text{Augmented}\;{\text{reality}}_{j}+\beta_{4n}.\text{Extra}\;{\text{Cost}}_{j}+\varepsilon_{\text{nj}}$$

with β_kn_ ∼ N (β_k_, σ_k_), MXL yields both a mean effect and a standard deviation of effects across the sample. Thus, MXL explicitly assumes a distribution of preference weights (coefficient β) across the sample, reflecting differences in preferences among respondents for the utility derived from the use of a personalized and connected prosthesis. β0 represents the Alternative Specific Constant (ASC), measuring the effect of the opt-out option.

Analyses were performed on all surgeons who answered to the survey, resulting in four sub-groups. These sub-groups were determined based on the main factors that influence surgical outcomes, namely age and experience [38]. In the case of France, where the average age of orthopedic surgeons is 50 and the median age is 51, surgeons under 50 were compared to those over 50 [39]. It is also recognized that a surgeon's experience is related to the volume of procedures performed per year. Therefore, we assumed that experienced surgeons perform over 51 procedures annually, based on the available data. These experienced surgeons are compared to those who perform fewer procedures [[40], [41], [42], [43]]. The p-value significance of the attributes and levels presented in choice experiments, are the main evaluation criteria used in this study.

This analysis is feasible as long as the minimum population size required is applied to each subgroup [44]. The sub-groups comparison was performed by using Pearson's Khi^2^ test on main variables. A p-value <0.05 was considered to be statistically significant. Multivariate logistic regression was used to identify relationships between variables.

ATI scale, which is graduated on six levels, was studied through mean, standard error, and Cronbach alpha coefficient. Sub-groups were compared by using the Wilcoxon test. A mean above four indicates a high or very high (over five) ATI and, in contrast, below, a low (three) or very low (two) ATI [45,46]. A Cronbach's alpha above 0.6 is considered satisfactory [47].

Data were analyzed by using R version 4.0.2.

## 3 Results

### 3.1 Respondents’ characteristics

A total of 90 male surgeons completed the questionnaire, providing their characteristics (Table 2). Most surgeons are over 50 years old, worked in public hospitals, and performed over 51 implants annually. They also mostly not used planning software, navigation systems, or robots in their surgeries. However, they expressed interest in adopting technological innovations, willing to extend surgical time by 10–20 min. Specifically, they were interested in using sensors to monitor patients, alongside standard consultations.

**Table 2:** Respondents’ characteristics. It is important to emphase that individuals under the age of 50 are distinct from those who performed fewer than 51 implants annually.

		Number	Percent (%)	IC 95 %
**Sex**	Male	90	100.0 %	0.0 %
	Female	0	0.0 %	0.0 %
**Age**	≤50 years old	34	37.8 %	10.2 %
	More than 50 year old	56	62.2 %	10.0 %
**Structure**	Public	54	60.0 %	10.0 %
	Private	36	40.0 %	10.1 %
**Number of prothesis per year**	≤50	34	37.8 %	10.0 %
	51–100	26	28.9 %	9.4 %
	Morethan 100	30	33.3 %	9.7 %
**Used of planification software**	Yes	43	47.8 %	10.3 %
	2D or 3D planning software	*24*	*55.8 %*	
	For 3D printed PSI (Personnalised Surgery Instruments)	*12*	*27.9 %*	
	Other (CT sheet - mixte)	*17*	*39.5 %*	
	No	47	52.2 %	10.3 %
**Navigation or robot**	Yes	28	31.1 %	9.6 %
	No	62	68.9 %	9.6 %
**Extra surgical time**	Yes	67	74.4 %	9.0 %
	Less than 10 min	*6*	*9.0 %*	
	More than 10 min	*44*	*65.7 %*	
	More than 20 min	*16*	*23.9 %*	
	Not defined	*2*	*3.0 %*	
	No	23	25.6 %	9.0 %
**Used of sensors**	Yes	66	73.3 %	9.1 %
	Yes, in addition to a standard consultation	*47*	*71.2 %*	
	Yes, in lieu of a follow-up consultation only	*2*	*3.0 %*	
	Yes, but only if the system issues an alert	*20*	*30.3 %*	
	No, it is not helpful	13	14.4 %	7.3 %
	I don't know	11	12.2 %	6.8 %

No significant relationships were found between categorical variables and age in terms of working structure, planning software use, navigation systems or robots use, extra surgical time, and sensor use. However, significance was found for the number of prostheses per year (Appendix A-1).

No significant relationships were found between categorical variables and the number of procedures performed per year with planning software, extra surgical time, and sensors. However, significance was observed for working structure, age, and use of navigation systems or robots (Appendix A-2).

Logistic regression analysis revealed that being over 50 years old was associated with a higher probability of performing more than 51 procedures per year and not utilizing data from sensors. No significant impact of the other variables was observed (Appendix B-1). Logistic regression analysis showed that performing over 51 procedures per year was associated with a higher likelihood of working in a public structure, being over 50 years old, and using navigation or robots. No significant impact of other variables was observed (Appendix B-2).

Surgeons showed a high affinity for technology interaction with no significant differences between subgroups (Appendix C).

### 3.2 Discrete choice experiment

Table 3 displays the estimated parameters of the MXL model analysis. Positive and significant coefficients are observed for use of technology-related attributes such as customized and connected implant, and AR. This suggests that surgeons find utility in using these attributes in their current practice. In terms of financial considerations, surgeons prefer a "+10 %" additional cost rather than "+25 %", indicating a willingness to incur limited expenses for implementing these technology-related attributes. Furthermore, the significant ASC indicates that surgeons prefer maintaining current practice despite desiring certain benefits from proposed technologies.

**Table 3:** Results from the random parameters mixed logit model. “ASC” stand for Alternative Specific Constant and “sd” for the standard deviation.

	All surgeons			Aged of 50 and less years old			Aged of more than 50 years old			50 and under procedures per year			51 and over procedures per year		
	Coeff	SE	p	Coeff	SE	p	Coeff	SE	p	Coeff	SE	p	Coeff	SE	p
ASC	0.503	0.069	***	0.389	0.089	***	0.641	0.108	***	0.264	0.110	*	0.649	0.089	***
Customized Implant															
Yes	0.829	0.069	***	1.193	0.105	***	0.262	0.091	**	0.949	0.116	***	0.853	0.088	***
No (Reference)	–	–		–	–		–	–		–	–		–	–	
Connected Implant															
Yes	0.383	0.062	***	0.563	0.088	***	0.124	0.088		0.546	0.106	***	0.339	0.078	***
No (reference)	–	–		–	–		–	–		–	–		–	–	
Augmented Reality															
Yes	0.848	0.070	***	0.889	0.098	***	0.712	0.099	***	0.933	0.120	***	0.770	0.088	***
No (Reference)	–	–		–	–		–	–		–	–		–	–	
Over-cost															
+10 %	0.580	0.064	***	0.670	0.089	***	0.505	0.093	***	0.590	0.106	***	0.602	0.081	***
+25 % (Reference)	–	–		–	–		–	–		–	–		–	–	
sd. Customized Implant	1.062	0.080	***	0.930	0.116	***	1.021	0.115	***	0.817	0.131	***	1.180	0.102	***
sd. Connected Implant	0.825	0.087	***	0.888	0.119	***	0.656	0.125	***	0.753	0.139	***	0.848	0.110	***
sd. Augmented Reality	0.850	0.085	***	0.916	0.112	***	0.722	0.128	***	0.789	0.146	***	0.849	0.104	***
sd. Over-cost	0.690	0.083	***	0.696	0.115	***	0.636	0.120	***	0.754	0.142	***	0.654	0.100	***

Signif. codes: 0 ‘***’ 0.001 ‘**’ 0.01 ‘*’ 0.05 ‘.’ 0.1 ‘’ 1.

Higher coefficients for attributes indicate greater utility derived from them. Among the attributes considered, AR and personalization show similar levels of utility, making them the most significant factors. On the other hand, the use of sensors has the lowest coefficient, indicating relatively lower importance compared to other attributes (Table 3).

All sub-groups preferred the current practice over the proposed technologies (positive and significant ASC).

Age-based subgroup analysis reveals that individuals over 50 do not significantly prefer sensors. Among those under 50, sensors are their least preferred option. AR is most preferred by those over 50 and ranks second for those under 50. Personalized prostheses are strongly preferred by those under 50, whereas personalized implants are the least preferred option for the other group. Annual procedure numbers-based subgroup demonstrates a distinct contrast between the two groups. Personalized prostheses provide the highest utility in both groups. Among surgeons performing fewer than 51 procedures annually, AR ranks equally with it, with sensor usage and a slight financial surcharge ranking second. In contrast, among surgeons performing over 51 procedures per year, AR is the second-highest preference, while sensors rank last. Finally, significant standard deviations in all groups indicate statistically significant heterogeneity in surgeons' preferences for all attributes.

## 4 Discussion

The analysis of our surgeon sample shows an experienced population, performing a significant number of procedures per year (mostly over 51) and spanning a wide age range. While they have a strong affinity for technology, they show limited inclination to integrate digital technology (e.g., preoperative software, navigation, robotics) into their surgeries. However, they are receptive to practice advancements and show willingness to allocate additional time and potentially adopt future sensors.

A detailed subcategory analysis reveals patterns beyond the correlation of annual procedures and age. Being over 50 years old negatively affects the utilization of sensor data, while performing over 51 procedures per year positively correlates with adopting navigation technology in a "public" work environment.

To assess surgeons' responses to implementing a new technological care chain, such as personalized and connected prostheses, we used the DCE method. Despite heterogeneity, all selected technological attributes positively influenced surgeons' preferences, with a willingness to adopt if costs were minimized. Surgeons over 50 were reluctant about sensor data, and overall, surgeons preferred their current practices. AR was favored, followed by personalization, while sensor utilization ranked lowest. Preferences varied by age and experience, with older surgeons preferring AR and more experienced surgeons favoring personalization.

The technologies in this new care chain are at different stages of maturity and usage, reflecting the preferences expressed. Personalization and assistive technologies are more advanced, while sensing is in its early stages.

The utilization of 3D planning software and 3D printing enables customized implants (CI) to reduce revision problems through individualized approaches. Accurate positioning and fit of knee prostheses are crucial for patient outcomes. Although currently used selectively, recent developments aim to promote wider adoption. Customized implants preserve the patient's unique geometry compared to off-the-shelf implants, but further improvements in mechanical properties are needed [[48], [49], [50]]. Surgeons in our study maintain high expectations for this technology [51]. Preference variation between young and experienced surgeons may be influenced by practical experience and the development stage of CI [9].

Proper positioning of CI is crucial due to individual patient morphology. Challenges in accurate positioning to ensure good functional outcomes that align with the anatomical needs have been reported. Nowadays, surgeons utilize navigation systems or robotics to replicate the resection plane from design software, aiding in achieving correct fit. Over the past two decades, the utilization of assisted technologies (Computer-Assisted Orthopedic Surgery [CAOS], robots, or PSI in Knee Arthroplasty has increased by 154 % from 2008 to 2015. For instance, robot utilization in hospitals increased by over 500 % from 2009 to 2013 [15]. Knee arthroplasty has the highest usage rate among other joints, with 18 % of knee surgeons reporting assisted technologies use in 2015 [12,52]. These proportion is expected to reach 32 % by 2032 [14]. CAOS and robots have demonstrated improved accuracy and precision in component positioning compared to PSI and conventional methods. However, no significant difference has been found in functional outcomes between them and conventional methods [52]. While current assisted technologies improve efficiency compared to conventional methods [52], they can be complex and bulky in the operating room, resulting in additional surgical time. On average, TKA takes an additional 15–25 min due to their use [15]. As part of the continuous effort to improve patient outcomes, AR has emerged as a promising innovation, streamlining workflow procedures, although limited studies with clinical results exist [17]. Indeed, existing systems often require surgeons to divert attention to a monitor, whereas AR partially addresses these limitations [53].

In our study, 31.1 % of surgeons utilized navigation systems or robots, with approximately 75 % requiring additional surgical time under 20 min. These findings align with Boylan's study [12], reporting a 29.2 % utilization rate of robotic assistance in New York hospitals in 2018. Surgeons' high preference for AR indirectly indicates their openness to user-friendly technologies.

Smart sensors, incorporated into implants to provide personalized data, show promise in monitoring post-operative and intraoperative parameters, assisting surgeons in problem detection and personalized rehabilitation [8]. However, challenges remain in areas like design, robustness, wireless communication, power, and biocompatibility. The use of connected implants in orthopedic surgeries (such as knee, hip, spine, and fractures) has been limited, with only around 100 patients involved, over the past few decades [18]. While some recent technologies have been adopted in routine practice, most evidence comes from clinical case studies [19]. Further advancements are needed to improve diagnostic capabilities, explore self-treatment options, ensure safety, data security, and reduce costs. Additionally, beyond technological aspects, it is crucial to enhance the safety of implants (including regulatory authorizations), ensure data security, and reduce costs. High costs may impede adoption in publicly funded healthcare systems [8]. Despite their potential, these technologies still have limitations and insufficient evidence. Surgeons' preferences reflect this.

Economic considerations affect indirectly choices and global adoption of technologies, depending on affordability and healthcare models [54]. For instance, in the United States, patients with private insurance and high-volume hospitals are more likely to undergo technology-assisted surgery, compared respectively to Medicare and Medicaid patients and lower-volume hospitals [12]. Larger hospitals can distribute technology costs and develop cost-effective care pathways [12,54]. In France, where the healthcare system determines costs, surgeons have limited control and moderate expense increases are expected to adopt these new technologies. However, a significant increase (+25 %) would negatively impact technology utilization.

This study has limitations. Firstly, surgeons were asked about technologies still under development, preventing pre-testing prior to answering the scenarios. Envisioning potential usage may have caused cognitive fatigue, possibly influencing choices towards the end of the 16 choice sets. The methodology was adapted to mitigate this effect. Secondly, survey respondents showed a tech-oriented population based on the ATI score. This introduces a potential selection bias, especially considering the small sample size, even if it meets the DCE criteria, in comparison to the practitioner population as a whole. However, this aligns with the "diffusion of innovation" theory, proposed by E. Rogers, new technologies and practices typically require endorsement from "early adopters" among surgeons [54].

Technology spread and adoption vary across countries due to different influencing factors [54]. Comparing relative preferences between French surgeons and those from other countries can provide insights into convergences and divergences in perceptions of new technologies.

In conclusion, our study reveals that, despite heterogeneity in responses, surgeons express a preference for utilizing a customized prosthesis through augmented reality, even at an additional measured cost. However, it appears that embedded sensor technology is currently less appealing to them, possibly due to its early-stage development.

## Data availability statement

The data have not been deposited into a publicly available repository. The data that has been used is confidential.

## Funding

This work benefited from state aid managed by the French National Research Agency under the France 2030 Program with reference N°. ANR-17-RHUS-0005 (Project Follow-Knee). No commercial funding was received for this study.

## CRediT authorship contribution statement

**Mathieu Le Stum:** Writing – review & editing, Writing – original draft, Software, Project administration, Methodology, Investigation, Formal analysis, Data curation, Conceptualization. **Arnaud Clave:** Writing – review & editing, Writing – original draft, Methodology, Investigation, Conceptualization. **Koffi Adzinyo Agbemanyole:** Writing – review & editing, Software, Formal analysis. **Eric Stindel:** Writing – review & editing, Validation, Supervision, Methodology, Investigation, Funding acquisition, Conceptualization. **Myriam Le Goff-Pronost:** Writing – review & editing, Writing – original draft, Validation, Supervision, Project administration, Methodology, Investigation, Conceptualization.

## Declaration of competing interest

The authors declare the following financial interests/personal relationships which may be considered as potential competing interestsStindel Eric reports financial support was provided by French National Research Agency. Stindel Eric reports a relationship with Ostesys SAS that includes: consulting or advisory. Clave Arnaud reports a relationship with ATF Lapée Médical that includes: consulting or advisory, speaking and lecture fees, and travel reimbursement. Clave Arnaud reports a relationship with Zimmer Biomet that includes: speaking and lecture fees. Clave Arnaud reports a relationship with CAOS France that includes: board membership. If there are other authors, they declare that they have no known competing financial interests or personal relationships that could have appeared to influence the work reported in this paper.

## Figures and Tables

**Table A:**
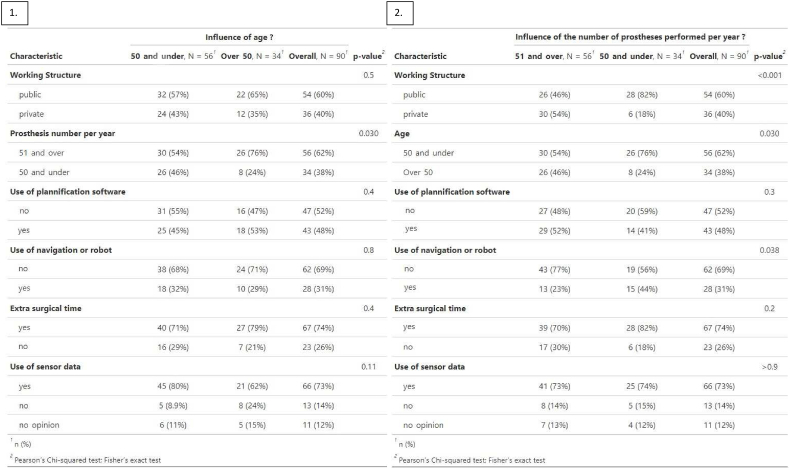
Comparison of sub-group. 1. Age. 2. Number of procedures performed per year

**Table B:**
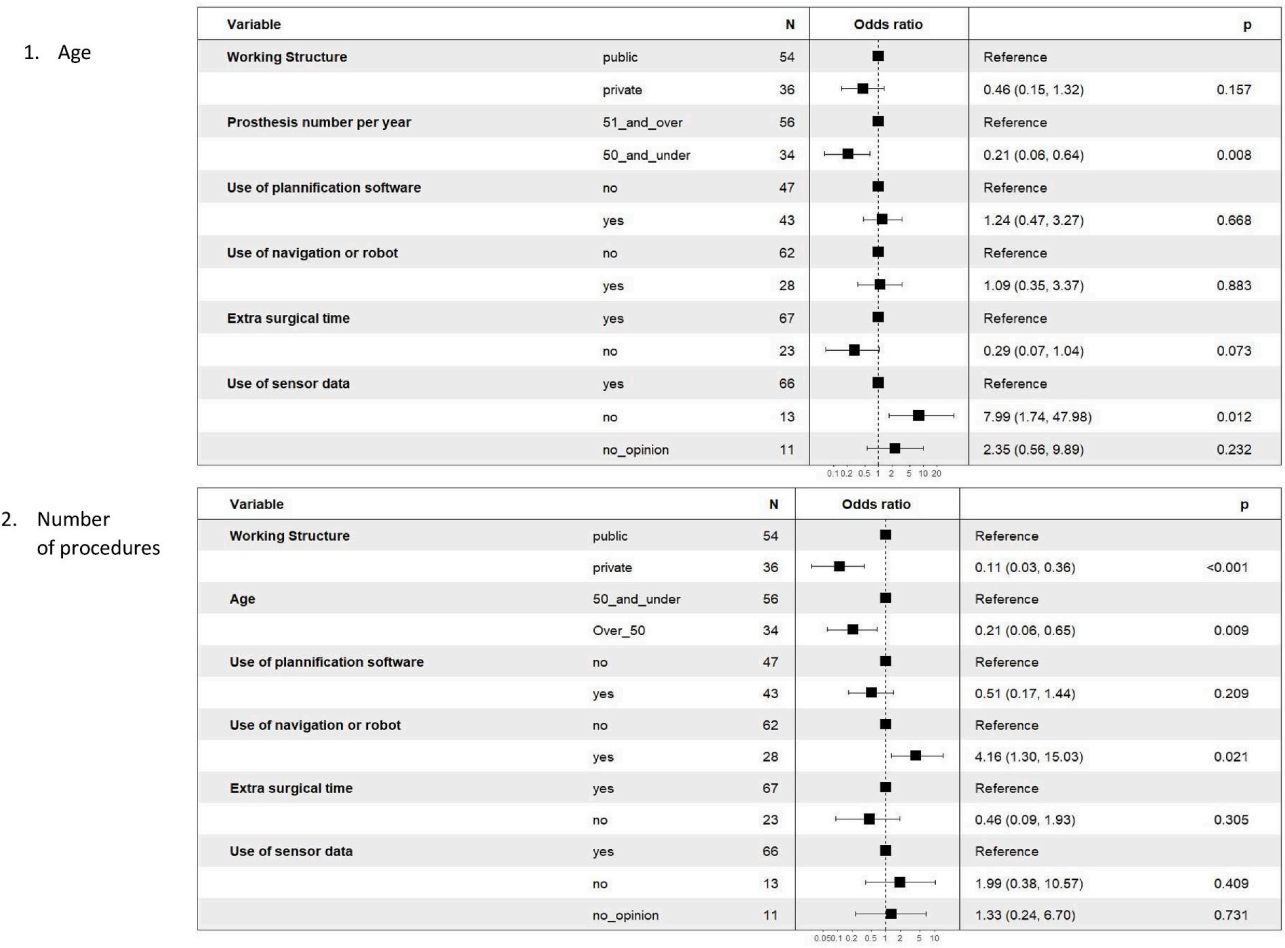
Logistic multivariate results (p = p-value). **1.** Age (as main variable). **2.** Number of procedures performed per year (as main variable).

**Table C:** Affinity for Technology Interaction (ATI) score. No significant differences were observed between subgroups (p > 0.05). Affinity for technology interaction are as follow: between 1 and 2, very low; between 2 and 3 low; between 3 and 4 medium; between 4 and 5 high; and between 5 and 6 very high.

	Mean	SD	Cronbach coefficient α
Global	4.58	1.32	0.65
50 years old and less	4.58	1.15	0.75
More than 50 years old	4.58	1.42	0.56
50 and under acts performed per year	4.62	1.29	0.77
51 and over acts performed per year	4.62	1.33	0.56
